# A commentary on ‘Long-term outcomes of intraoperative chemotherapy with 5-FU for colorectal cancer patients receiving curative resection (IOCCRC): a randomized, multicenter, prospective, phase III trial’

**DOI:** 10.1097/JS9.0000000000001747

**Published:** 2024-05-29

**Authors:** Zongbao Yin, Gang Zhang

**Affiliations:** a Department of Emergency, Central South University Xiangya School of Medicine Affiliated Haikou Hospital; b Department of Gastrointestinal Surgery, Central South University Xiangya School of Medicine Affiliated Haikou Hospital, Haikov, Hainan Province, People’s Republic of China

*Dear Editor,*

We read with great interest the article titled ‘Long-term outcomes of intraoperative chemotherapy with 5-FU for colorectal cancer patients receiving curative resection (IOCCRC): a randomized, multicenter, prospective, phase III trial’^1^. The authors discovered that intraoperative chemotherapy showed no significant extra prognostic benefit in total colorectal cancer patients who underwent radical surgical resection; however, in colon cancer patients with abnormal pretreatment serum CEA (carcinoembryonic antigen) levels, intraoperative chemotherapy could improve long-term survival. In general, it is a well-designed study. However, we would like to address some questions concerning the content of the study.

Firstly, tumors involving the serosal layer are more susceptible to peritoneal implantation compared to early-stage tumors and those completely within the mesentery. A stratified analysis by T stage and tumor location would be valuable in understanding these dynamics more clearly. Secondly, there appears to be a discrepancy in Table 1 regarding surgical procedures. While the table lists 170 cases of rectal cancer, the combined total of Anterior resection, Abdominoperineal resection, and Hartmann’s operation is 240 cases. Could this discrepancy be explained by including some patients with sigmoid colon cancer who also underwent Anterior resection? Clarification on this point would help ensure the accuracy of the data presented and its interpretation.

These adjustments are crucial for ensuring the accuracy and relevance of the study’s outcomes within the context of neoadjuvant therapy. We believe that addressing these concerns will contribute significantly to the robustness of the study’s findings.

## Ethical approval

For our study, ethical approval has been granted by the ethics committee of Central South University Xiangya School of Medicine Affiliated Haikou Hospital.

## Consent

The letter does not involve patients or volunteers.

## Source of funding

Not applicable.

## Author contribution

G.Z.: revising the manuscript; Z.Y.: submission process. All authors contributed to the writing of this letter.

## Conflicts of interest disclosure

There are no conflicts of interest.

## Research registration unique identifying number (UIN)

Not registered for the type of study.

## Guarantor

Gang Zhang is fully responsible for the work and/or the conduct of the study, has access to the data, and controls the decision to publish.

## Data availability statement

The letter does not have any data.

## Provenance and peer review

Not applicable.
